# Efficacy of morphine versus fentanyl patient-controlled analgesia for postoperative pain management in colorectal surgery

**DOI:** 10.5339/qmj.2025.7

**Published:** 2025-02-23

**Authors:** Adnan Saad Eddin, Hazem Selim, Roaa Suleiman, Jeena Thomas

**Affiliations:** ^1^Department of Anesthesiology, Hamad Medical Corporation, Doha, Qatar *Correspondence: Adnan Saad Eddin. Email: saadeddinmd@gmail.com

**Keywords:** Patient-controlled analgesia, postoperative pain management, fentanyl, morphine, colorectal surgery

## Abstract

**Introduction:**

Postoperative pain management is crucial for recovery from surgery. Patient-controlled analgesia (PCA) with morphine and fentanyl are commonly used, but their comparative efficacy remains uncertain. This study aims to evaluate opioid consumption and pain control in patients receiving PCA morphine versus PCA fentanyl after colorectal surgery.

**Methodology:**

A retrospective analysis of adult patients undergoing elective colorectal surgery was conducted. Patients were divided into two groups based on PCA morphine or PCA fentanyl use. Outcomes measured were opioid consumption in morphine equivalents, numerical pain scores expressed as Numerical Rating Scale (NRS), patient demand, and side effects within the first 48 hours postoperatively.

**Results:**

Of 370 patients screened, 152 met the inclusion criteria. No significant differences were found in total opioid consumption (median: 38 vs. 28.5 mg, *p* = 0.095), patient demand (median: 46.5 vs. 35, *p* = 0.156), or NRS (median: 4 vs. 3.5, *p* = 0.348). Side effects were comparable between groups. Subgroup analysis revealed higher opioid consumption and demand in females taking fentanyl compared to morphine. Age was negatively correlated with pain-related outcomes, and smokers showed higher opioid consumption and higher pain scores.

**Conclusions:**

PCA morphine and fentanyl provide similar postoperative pain relief in colorectal surgery patients, with no significant differences in opioid consumption or side effects. Female patients may respond better to morphine, and age and smoking status significantly influence pain management outcomes. Further prospective studies are recommended to better define these findings and inform postoperative pain strategies.

## INTRODUCTION

Postoperative pain is a common complication following surgery and can significantly impact patient recovery, satisfaction, and overall outcome.^1^ Colorectal surgery poses significant challenges for postoperative pain management due to the nature and extent of the surgical intervention. Patient-controlled analgesia (PCA) has emerged as the preferred method for managing postoperative pain. It allows patients to self-administer analgesic agents within the preset limits, thereby achieving a balance between pain relief and side effects. Morphine and fentanyl are the most commonly used agents in PCA, each with its distinct pharmacological profile and implications for patient care.^2^ PCA devices are typically programmed to deliver controlled doses of opioids to ensure that the patient receives pain relief without the delay of nurse administration and to reduce the risk of both underdosing and overdosing of opioids.

Fentanyl is a highly lipid-soluble synthetic opioid that crosses the blood–brain barrier rapidly and reaches a peak effect within 5 minutes of administration. It is considered to be 50–100 times more potent than morphine.^3,4^ Its duration of action is relatively short, generally around 30–60 minutes.^5^ Morphine, on the contrary, is a less potent, naturally occurring hydrophilic opioid with a slower onset of action, typically peaking after 10–20 minutes of administration, and a longer duration of action that can last up to 4 hours.^6^

The comparative efficacy and safety of morphine and fentanyl in postoperative pain management is still debatable. Several studies have attempted to compare the efficacy of PCA morphine and PCA fentanyl postoperatively, but they report conflicting results. Some found no difference between morphine and fentanyl,^7,8^ while others found evidence of superiority of morphine.^9,10^

Furthermore, there is a lack of research comparing fentanyl and morphine PCA specifically in patients who have undergone colorectal surgery. This retrospective study aims to compare opioid consumption, expressed in morphine equivalent dose (MED), between PCA morphine with PCA fentanyl in the postoperative pain management of patients who underwent colorectal surgery. The findings of this study will provide a better understanding of the optimal analgesic choice for PCA and potentially inform postoperative pain management strategies.

## METHODOLOGY

### Study design and participants

This study included patients who underwent elective colorectal surgery and received PCA fentanyl or morphine for at least 48 hours postoperatively between January 2022 and May 2023. The acute pain service registry and electronic patient records were retrospectively reviewed to identify patients who met our inclusion criteria: adult patients ( ≥ 18 years) who underwent elective colorectal surgery and received PCA with either morphine or fentanyl immediately postoperatively.

Exclusion criteria included allergy to morphine or fentanyl, chronic opioid use or abuse, pre-existing chronic pain conditions, emergency surgery, renal impairment, hepatic impairment, cognitive impairment, ICU (Intensive Care Unit) admission after surgery, PCA treatment for less than 48 hours postoperatively, administration of regional anesthesia or analgesia, device settings different from standard practice, switching from one PCA agent to another within the first 48 hours after surgery, and incomplete documentation of PCA settings, numerical pain scores expressed as Numerical Rating Scale (NRS), total opioid consumption, or side effects in patient records.

### Outcomes

Data were collected on opioid consumption, numerical pain scores, PCA device information including lockout interval, basal infusion, bolus settings, as well as patient demand and boluses delivered. Additional data were collected on the incidence of nausea and vomiting, the incidence of dizziness, the medical history, and the medications received intra- and postoperatively. All data collected were limited to the first 48 hours postoperatively. The numerical pain score was measured by asking the patients to rate their pain at the time of measurement by selecting a number from 0 to 10, where 0 represents no pain and 10 represents worst possible pain.

The two groups were compared in terms of the total opioid consumption, expressed in MED, during the first 48 hours postoperatively. Fentanyl is considered to be 50–100 times more potent than morphine. In our practice, 20 mcg of fentanyl is considered equivalent to 1 mg of morphine, and this equipotency was considered in this analysis. In addition to the opioid received through PCA, some patients required additional rescue opioid. The most commonly used rescue medication is morphine, and less frequently fentanyl. According to the hospital protocol, rescue opioid is administered when the numerical pain score is higher than 3.

The two groups were also compared for patient demand (the number of times the patient pressed the PCA button), average numerical pain scores (measured every 24 hours), incidence of dizziness, and occurrence of nausea and vomiting, all in the first 48 hours postoperatively. The associations of age, gender, and smoking history with total opioid consumption and numerical pain scores were examined. The lockout intervals were also compared.

### Statistical analysis

Continuous variables were summarized according to their distribution using either median and interquartile range (IQR) or mean and standard deviation. Categorical variables were presented as counts and percentages. Normality testing was carried out graphically and statistically using the Shapiro–Wilk test.

Comparison between the groups for continuous outcome variables was performed using the Mann–Whitney U test (also known as the Wilcoxon rank-sum test) or the t-test, depending on the distribution of the variable. The Mann–Whitney U test was used for total opioid consumption, patient demand, and numerical pain scores, while the t-test was used for the incidence of nausea and vomiting and the incidence of dizziness. The associations were examined using the chi-square test for categorical variables and the Spearman rank correlation test for continuous variables.

All statistical analyses were performed using IBM SPSS statistical software (version 29.0.2.0). The statistical significance limit was set at a *p* value of ≤ 0.05.

## RESULTS

The baseline characteristics of the cohort for the study are summarized in Table 1. A total of 370 patients who underwent colorectal surgery and received either PCA fentanyl or PCA morphine for postoperative pain management were screened for eligibility. Of these, 152 patients were eligible for inclusion: 80 patients received fentanyl and 72 patients received morphine. Male patients represented the majority of the sample (71.05 %). The mean age of the participants was 50.3 ± 14.05 years. No significant differences were observed between the groups in terms of baseline characteristics. None of the patients received a basal infusion, and the bolus dose for all patients was set at 20 mcg for fentanyl and 1 mg for morphine. The lockout interval was set at 5 minutes for 94.1% of patients and 10 minutes for 5.9% of patients, with no statistically significant difference found between the groups.

There was no statistically significant difference in total opioid consumption between the PCA fentanyl and PCA morphine groups within the first 48 hours postoperatively (median: 38 MED vs. 28.5 MED, p <  0.095) (Table 2).There was also no significant difference in patient demand (median: 46.5 times vs. 35 times, *p* = 0.156) and numerical pain scores (median: 4 vs. 3.5, *p* = 0.348) (Table 2).

A subgroup analysis based on gender was performed. The analysis revealed that in the female subgroup, total opioid consumption (median: 44 MED for fentanyl vs. 19.5 MED for morphine, *p* = < 0.001) and patient demand (median: 55 times for fentanyl vs. 23 times for morphine, *p* = 0.002) were higher in the fentanyl group than in the morphine group. There were no significant differences in the outcomes in the male subgroup. To further define the association, males and females were compared within each PCA group, which showed a statistically significant difference in opioid consumption between males and females in the PCA morphine group (median: female 19.5 MED vs. male 23 MED, *p* = 0.009). On the contrary, this difference was not observed in the PCA fentanyl group (median: female 44 MED vs. male 37 MED, *p* = 0.11).

The two groups were compared for the incidence of nausea/vomiting and the incidence of dizziness, which showed no differences between the groups in the incidence of nausea/vomiting (fentanyl 17.5% vs. morphine 12.5%, *p* = 0.390) and the incidence of dizziness (fentanyl 10% vs. morphine 5.6%, *p* = 0.310). The results were consistent across males and females.

**Figure 1.**
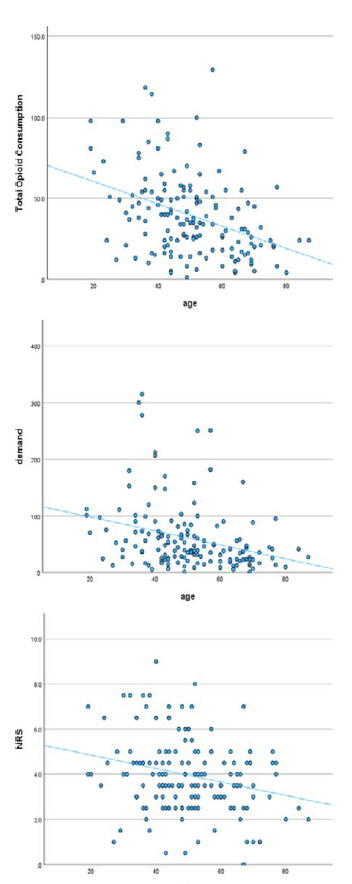
Scatter plots for the correlation between pain-related outcomes and age.

A negative correlation was observed between age and each of the pain-related outcomes (total opioid consumption, patient demand, and numerical pain scores) (Table 3, Figure 1).

The role of gender and smoking on pain outcomes was examined. No correlation was found between gender and total opioid consumption (*p* = 0.395), patient demand (*p* = 0.292), or numerical pain scores (*p* = 0.204) in the first 48 hours postoperatively. A significant difference in the incidence of nausea and vomiting was observed between genders, with 25% of females and 11.1% of males experiencing symptoms (*p* = 0.03). Additionally, the incidence of dizziness was significantly higher in females than in males (18.2% vs. 3.7%, *p* = 0.003).

A significant difference was observed between smokers and non-smokers in total opioid consumption, patient demand, and numerical pain scores (Table 4).

No significant association was found between the lockout interval and total opioid consumption (*p* = 0.696), total demands (*p* = 0.512), or numerical pain scores (*p* = 0.808). These results suggest that a lockout interval of 10 minutes is as effective as 5 minutes.

## DISCUSSION

Our study showed that pain-related outcomes (total opioid consumption, patient demand, and numerical pain scores) did not differ between patients who received PCA morphine and those who received PCA fentanyl. There was also no statistically significant difference in the incidence of nausea, vomiting, and dizziness between the two groups.

Previous studies have reported conflicting results. Some studies have suggested that PCA morphine is superior to PCA fentanyl. In a retrospective study of patients undergoing liver resection, PCA morphine was found to be superior in terms of total opioid consumption and patient demand, but not in terms of numerical pain scores.^9^ Another prospective non-randomized study comparing the two agents in patients undergoing uterine fibroid embolization found that morphine provided better numerical pain scores than fentanyl.^10^ However, it did not provide a clear comparison for total opioid consumption. A randomized trial comparing fentanyl to morphine in patients undergoing cesarean section found no differences in terms of visual analog scale. However, patients in the fentanyl group required more rescue doses than those in the morphine group.^8^

On the contrary, a prospective, randomized, double-blind study comparing morphine, fentanyl, and remifentanil in patients undergoing cardiac surgery found no differences between morphine and fentanyl in terms of visual analog scale, patient demand, and boluses delivered, despite using fentanyl boluses of 10 mcg, which is 50% of the fentanyl bolus doses used in our study.^11^ In another double-blind, randomized trial that compared PCA morphine, fentanyl, and pethidine postoperatively, no difference was found between morphine and fentanyl in terms of total opioid consumption, patient demand, and visual analog scale.^12^

In terms of the side effect profile, this study found a comparable incidence of nausea and vomiting and dizziness between the two groups. These findings were consistent with those reported in the literature.^7,8,12,13^ One of the studies mentioned above reported a higher incidence of nausea in the morphine group, although the finding was not statistically significant.^10^

The study found no difference between the lockout intervals used (5 minutes and 10 minutes) in terms of pain-related outcomes and the side effect profile. This finding is consistent with the literature that compared different lockout intervals. In one study, researchers compared lockout intervals of 7 and 11 minutes for morphine, and 5 and 8 minutes for fentanyl, and found no differences in pain relief and side effects.^14^ In another study, researchers compared different dosages and lockout intervals of morphine and found no differences between the groups in terms of total opioid consumption, analgesia, and the incidence of side effects.^15^ These results suggest that clinicians may have flexibility in selecting the lockout interval for PCA without compromising pain control or increasing the risk of opioid-related side effects.

A significant negative correlation was found between age and pain-related outcomes (total opioid consumption, patient demand, and numerical pain scores). This correlation is explained by physiological changes in the elderly. Aging is associated with a decline in renal and hepatic function, which can affect the metabolism and clearance of opioids. Older adults may have slower metabolism of opioids, leading to prolonged effects and an increased risk of accumulation and toxicity.^16^ Additionally, an increase in body fat and a decrease in lean body mass and total body water are common in older adults. These changes can affect the distribution of lipophilic drugs such as opioids, potentially altering their efficacy and side effect profile.^17,18^ Furthermore, age-related changes in the nervous system, such as reduced nerve fiber density and changes in neurotransmitter levels, may affect pain perception.^19^

The subgroup analysis revealed that females responded better to morphine than males, while both responded equally to fentanyl. Two of the studies comparing PCA fentanyl and PCA morphine were aimed exclusively at females,^8,10^ and morphine was superior to fentanyl in both studies. It is unclear what explains the differences in response to morphine, but not to fentanyl, between males and females, and this requires further research. Research has consistently shown that men and women experience and report pain differently, with women generally having lower pain thresholds and reporting higher pain scores than men.^20^ Sex hormones, particularly estrogen, are known to influence pain pathways, alter pain perception, and affect responses to certain classes of drugs, including opioids.^24^ Interestingly, however, some studies have not suggested significant sex differences in the postoperative period. For example, a reanalysis of a randomized clinical trial found no differences in pain severity, pain perception, or postoperative opioid use between men and women after lumbar spine sequestrectomy.^25^ Another study concluded that men required more morphine than women in the postoperative period following abdominal surgery.^26^

Smoking was found to be associated with higher rates of opioid consumption, higher patient demand, and higher pain scores. This is consistent with the findings from the literature that chronic tobacco use is associated with increased postoperative pain sensitivity.^27–30^ There are multiple mechanisms that explain the increased pain perception and analgesic requirements in smokers. These include altered pain processing due to receptor desensitization and upregulation, nicotine withdrawal, neuroendocrine changes, pain-induced reinforcement of smoking behaviors, impaired wound healing, decreased tissue oxygenation, and psychological factors.^31^

Our study is limited by its retrospective design. A prospective randomized trial would better define the differences between fentanyl and morphine in the PCA context and may limit the effects of unmeasured confounders. Additionally, our findings were derived from a single tertiary hospital, which may limit its generalizability to other clinical settings. Furthermore, our study focused on pain intensity and rescue analgesic requirements, which may not fully capture the quality of postoperative pain management. Due to limited data availability in the records analyzed, our study did not compare the trends in opioid consumption and pain scores, which could have provided better insights into the dynamic differences between PCA fentanyl and morphine. The study also focused on elective colorectal patients only, a decision made to control for the confounders. Additionally, the study did not compare the effect of the two PCA regimens on different colorectal pathologies. However, different surgical procedures can have different pain profiles. The study was powered to detect the differences in total opioid consumption, and the other outcomes should be carefully interpreted. Finally, our study only compared two PCA regimens and does not provide comparisons with other regimens.

## CONCLUSION

The study evaluated the efficacy and safety of PCA using morphine compared to fentanyl postoperatively in patients undergoing colorectal surgery. The analysis revealed no significant differences between PCA morphine and PCA fentanyl in terms of pain-related outcomes. Additionally, the incidence of side effects such as nausea, vomiting, and dizziness was comparable between the two groups. Subgroup analysis indicated that female patients had higher opioid consumption and patient demand when using fentanyl compared to morphine, while no significant differences were observed in male patients. Furthermore, a significant negative correlation was found between age and pain-related outcomes. Smoking was associated with higher opioid consumption, higher patient demand, and higher pain scores.

### List of abbreviations

[Table tbl5]

### Competing interests

The authors have no conflicts of interest to declare.

### Ethical approval

The study was approved by the Medical Research Center (MRC) at Hamad Medical Corporation (MRC-01-23-628).

## Figures and Tables

**Table 1.** Patient characteristics.

Characteristics	*N*	Fentanyl	Morphine	*p*
Number of patients	152	80	72	
Gender, *n* (%)				0.309
Female	44 (28.6%)	26 (32.5%)	18 (25%)	
Male	108 (71%)	54 (67.5%)	54 (75%)	
Age, years (mean (SD))	50.4 (14)	49.64 (13.89)	51.18 (14.27)	0.452
BMI, kg/m^2^ (median (IQR))	26.42 (6.99)	26.5 (8)	26.1 (6.3)	0.833
Surgery technique, *n* (%)				0.169
Laparoscopic	108 (71.1%)	53 (66.2%)	55 (76.4%)	
Open	44 (28.9%)	27 (33.8%)	17 (23.6%)	
Fentanyl IO, *n* (%)	56 (36.8%)	24 (30.0%)	32 (44.4%)	0.065
Remifentanil IO, *n* (%)	123 (80.9%)	69 (86.2%)	54 (75.0%)	0.078
Morphine IO, *n* (%)	145 (95.4%)	75 (93.8%)	70 (97.2%)	0.308
Paracetamol IO, *n* (%)	142 (93.4%)	76 (95.0%)	66 (91.7%)	0.408
NSAID IO, *n* (%)	102 (67.1%)	52 (65.0%)	50 (69.4%)	0.560
Antiemetic IO, *n* (%)	141 (92.8%)	74 (92.5%)	67 (93.1%)	0.895
NSAID PO, *n* (%)	133 (87.5%)	70 (87.5%)	63 (87.5%)	1.000
Paracetamol PO, *n* (%)	152 (100%)	80 (100.0%)	72 (100.0%)	
Antiemetic PO, *n* (%)	58 (38.2%)	29 (36.2%)	29 (40.3%)	0.610
Lockout Interval, *n* (%)				0.385
10 minutes	9 (5.9%)	6 (7.5%)	3 (4.2%)	
5 minutes	143 (94.1%)	74 (92.5%)	69 (95.8%)	
Required rescue medication, *n* (%)	21 (13.8%)	12 (15%)	9 (12.5%)	0.656
Smoking, *n* (%)	21 (13.8%)	7 (8.8%)	14 (19.4%)	0.056
Alcohol, *n* (%)	9 (5.9%)	6 (7.5%)	3 (4.2%)	0.302

SD: standard deviation, BMI: body mass index, IQR: interquartile range, IO: intraoperative, NSAID, non-steroidal anti-inflammatory drugs; PO: postoperative,

**Table 2.** Mann–Whitney tests of dependent variables.

	Fentanyl		Morphine		
Variables	Median	IQR	Median	IQR	*p*
Total opioid consumption	38	30	28.5	35	0.095
Patient demand	46.5	52	35	56	0.156
Numerical pain score	4	2.4	3.5	1.5	0.348

**Table 3.** Effect of age on opioid consumption and pain scores.

**Variables**	Correlation coefficient^*^	*p*
Total opioid consumption	-0.377	< 0.001
Patient demand	-0.356	< 0.001
Numerical pain score	-0.214	0.008

*The Spearman correlation coefficient was used.

**Table 4.** Effect of smoking on opioid consumption and pain scores.

**Variables**	**Smoker (median)**	**Non-smoker (median)**	*p* ^*^
Total opioid consumption	49	34	0.001
Patient demand	52	38	0.002
Numerical pain scores	5	3.5	0.005

*Independent-samples Mann–Whitney U test.

**Table tbl5:** 

BMI	Body Mass Index
ICU	Intensive Care Unit
IO	Intraoperative
IQR	Interquartile Range
MED	Morphine Equivalent Dose
NRS	Numerical Rating Scale
NSAID	Non-Steroidal Anti-Inflammatory Drugs
PCA	Patient-Controlled Analgesia
PO	Postoperative
SD	Standard Deviation
SPSS	Statistical Package for the Social Sciences
